# Use of Electronic Health (eHealth) Among Saudi Type 2 Diabetic Patients and Its Association With Their Diabetic Self-Management: A Cross-Sectional Study

**DOI:** 10.7759/cureus.13882

**Published:** 2021-03-14

**Authors:** Asmaa Abdel Nasser, Razan M Alzahrani, Ahmed N Ghandoura, Intessar Sultan

**Affiliations:** 1 Medical Education, Faculty of Medicine, Suez Canal University, Ismailia, EGY; 2 Medicine, Ibn Sina National College for Medical Studies, Jeddah, SAU; 3 Internal Medicine, Ibn Sina National College for Medical Studies, Jeddah, SAU

**Keywords:** electronic health, ehealth, type 2 dm, health care visits, self-care management

## Abstract

Background

Type 2 diabetes mellitus (T2DM) is a prevalent, chronic, non-communicable disease that requires continuous multidisciplinary health care. Electronic health (eHealth) refers to “the transfer of health information resources and health care services using different electronic platforms.” This may have an effect on diabetes self-management (DSM).

Objectives

This study aimed to identify the use of eHealth among patients with T2DM as well as its association with DSM.

Method

An analytical cross-sectional study was conducted online using a newly adapted three-part questionnaire using Google Forms through different social media platforms. A total of 2,228 adult Saudi T2DM patients from different provinces were selected based on the non-probability voluntary response sampling technique. The survey included demographic, clinical, and eHealth data, and diabetic self-care management.

Results

The study results revealed an average DSM score of 5.2/10, and 74.1% were receiving diabetes care at primary health care centers. Of these, 87.1% used eHealth, mainly through Google (55.7%) and other social media (12.9%), and were satisfied with the quality of health care (70.4%). Moreover, 82% wanted to discuss the eHealth information with their physicians, but some (34.5%) had no online access to them. eHealth dependency was 44.2% and was associated with a lower mean DSM (5.6 vs. 5.3; p = 0.000) with significantly lower health care use (6.7 vs. 5.6; p = 0.000) and glucose management (4.7 vs. 4.0, p=0.000) compared to the independent group. The DSM total score was a significant predictor of eHealth dependency (OR: 1.022; 95% CI: 1.006-1.039; p = 0.007).

Conclusion

Most Saudi T2DM patients with an average DSM use different eHealth resources and are satisfied with their quality. Dependency to eHealth is significantly associated with lower DSM, especially for health care use and glucose management, a finding that could affect patient outcomes. Still, patients need to communicate with their physicians in person who should have different options for remote consultation, such as telemedicine, to support their patients.

## Introduction

Type 2 diabetes mellitus (T2DM) is a prevalent complex, chronic, non-communicable disease that requires continuous multidisciplinary health care. Ongoing diabetes self-management (DSM) education is crucial for preventing acute complications, reducing the risk of long-term complications, and improving patient outcomes [1]. Proper DSM support should follow the steps of evidence-based medicine and be initiated and monitored by skilled health professionals in a consistent manner. It should follow well-designed programs that address all patient aspects and challenges [2].

The term eHealth (electronic health) refers to “the transfer of health information resources and health care services using different electronic platforms” using the Internet [3]. It is currently an area of evolution with a possible impact on DSM. eHealth can support the exchange of medical information, improve communication, and provide patient education [4]. It shows an exceptional impact on diabetic patients who are in need of continued information and self-care, as well as frequent monitoring and regular follow-up visits [5,6]. Understanding the interaction between eHealth and health care utilization among patients with diabetes is of great importance for constructing evidence-based regulations for better health outcomes [7]. The interaction of eHealth with diabetes care has some positive and negative impacts. Researchers have reported an increasing number of diabetic patients using eHealth with potential benefits and risks of diabetes care [8-10]. eHealth can provide essential information before or after the hospital visit or direct patients to seek medical consultation [11]. eHealth risks include retrieving inaccurate, incomplete, and non-evidence-based information, especially if the users are of low health literacy levels or if there is a lack of direct communication with their health care providers [12-14]. The risks could also include spreading rumors or misinformation and endangering patients’ privacy [15]. Still, there is a need for more research from different cultural and economic settings to outline the benefits and risks of using eHealth among diabetic patients [7].

Most (83.9%) of the population with diabetes mellitus (DM) in the region are living in low- or middle-income countries. Saudi Arabia and Kuwait are among the countries with high diabetes prevalence (raw diabetes prevalence of 17.6% and 14.3%, respectively). The countries with the largest number of adults with diabetes are Egypt (7.8 [3.8-9.0] million), Pakistan (7.0 [5.1-10.0] million), and Iran (4.6 [3.6-6.3] million) [16]. One study in Saudi from two large public university hospitals in Riyadh reported the use of eHealth by almost one-quarter of the T2DM patients and that eHealth information represented the second source of their self-care data after consultation of their physicians [17]. Therefore, the aim of this study was to identify the use of eHealth among Saudi T2DM patients as well as its association with their DSM.

## Materials and methods

Study context and design

An analytical cross-sectional study was conducted by Ibn Sina National College for Medical Studies, Jeddah, Saudi Arabia, among Saudi T2DM patients. The study duration was six months (October 2019 to March 2020). The inclusion criteria included Saudi adults diagnosed with T2DM for at least one year up to five years. Patients with incomplete responses were excluded from the study. The sample size for the study was set at 2,200 participants, with a 95% confidence level and 2.75 error of precision.

Data collection methods

**Figure 1:**
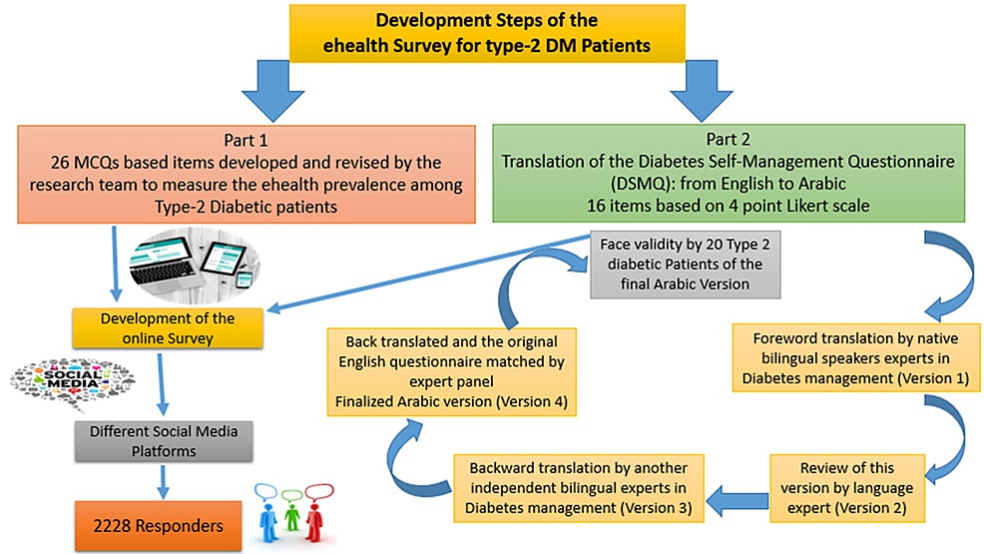
Steps in the development of the eHealth dependency online survey

A newly adapted three-section online questionnaire to measure T2DM eHealth dependency was sent via Google form through different electronic platform applications to T2DM patients who were selected using the non-probability voluntary response sampling technique (Figure 1). The first section of the questionnaire included the demographic data of the participants (age, gender, and regional area). The second section addressed the use of eHealth, which consisted of 26 closed questions that were developed and modified in accordance with the research objectives. The third section collected data on diabetes duration, treatment, frequency of visits, awareness of diabetes care (questions about type and number of point-of-care lab tests during follow-up), concerns, and satisfaction. Then, eHealth methods and applications were used to follow up and assess its association with changes in the patients' diabetic care. Patients who agreed to replace the physician consultation with eHealth modalities were considered as the eHealth-dependent group.

The participants were also asked to respond to an Arabic version of the Diabetes Self-Management Questionnaire (DSMQ). The DSMQ is a reliable and valid instrument for evaluating diabetes self-care behaviors in association with glycemic control [18]. The questionnaire was translated into Arabic using a standardized forward and backward translation procedure, as recommended by Bradley [19]. Participants were rated using the 16 items of the DSMQ, describing specific self-care behaviors according to their own diabetes control during the last one to five years. Rating is done based on a four-point Likert scale (from 0 “does not apply to me” to 3 “applies to me very much”; a neutral response option is not available in order to extract more specific results plus avoid of central tendency effect). The questionnaire contained 16 items: five for glucose management, four for dietary control, three for physical activity, three for health-care use, and one general question item. Of these 16 items, seven were formulated positively and nine were inversely formulated to calculate the total summation score. Then, each score was transformed into a score out of 10 (raw score/maximum score x10). Therefore, the higher the score, the better the self-management of diabetes.

Ethical considerations

Ethical approval for this study was obtained from the Ibn Sina National College Research and Ethics Committee in accordance with the Declaration of Helsinki for human studies [20]. During the online survey, the participants were informed about the purpose of the study and their right to refuse participation. Ethical conduct was maintained during data collection and throughout the research. Participation in the study was voluntary, and the confidentiality of the participants was maintained as the questionnaire was provided anonymously.

Statistical analysis

The collected data were coded and analyzed by a computer using a database software program, Statistical Package for Social Science (SPSS) Version 20 (IBM Corp., Armonk, NY, USA). Quantitative variables are expressed as numbers and percentages. The DSM individual and total scores were calculated out of 10, and their median results were used to construct Figure 1 using excel sheets and compare between the two groups eHealth dependency using the independent samples non-parametric Kruskal-Wallis U test. Logistic regression (binary regression) analysis was used to detect the significant predictors of eHealth dependency, and the exponential B with its 95% confidence interval (CI) was considered the odds ratio (OR). The results were considered statistically significant when the two-tailed p-value was less than 0.05.

## Results

In this study, 2228 T2DM patients were included from different provinces and different age groups, with 44.6% aged 45-55 years. Approximately 40% had diabetes for more than five years. The patients were treated with different types of antidiabetic therapy (40.1% insulin). Their diabetes care was mainly provided by governmental primary health care centers (71.6%). Only 48.3% were confident about their diabetes management, and 38.6% were aware of their diabetes care. Many (83.4%) patients managed their lifestyle, and 27.6% preferred self-medication. Only 17.7% were unsatisfied with their care provided by physicians, 28.9% had no physician consultation within the last 12 months, and 36.9% used to see their physician every three months (Table 1).

**Table 1:** Characteristics of the participating diabetic patients (n = 2,228)

Characteristics		N	%
Saudi Arabia residency	North	314	14.1%
	West	352	15.8%
	East	486	21.8%
	Central	534	24.0%
	South	542	24.3%
Age (years)	18-25	35	1.6%
	25-35	598	26.8%
	35-45	601	27.0%
	45-55	994	44.6%
Duration of diabetes	1 year	564	25.3%
	2 years	281	12.6%
	3 years	253	11.4%
	4 years	251	11.3%
	≥5 years	879	39.5%
Smoking		659	29.6%
Treatment of diabetes	Lifestyle	468	21.0%
	Low carb	180	8.1%
	Oral drugs	687	30.8%
	Insulin	893	40.1%
Site of diabetes care	Private	632	28.4%
	Governmental	1596	71.6%
Frequency of follow-up	Monthly	446	20.0%
	Every 3 months	823	36.9%
	Every 6 months	959	43.0%
Physician consultation within 12 months	No	643	28.9%
	Yes	1585	71.1%
Satisfaction with physicians' care	Not satisfied	395	17.7%
	Satisfied	1340	60.1%
	Very Satisfied	493	22.1%
Lifestyle management	No	370	16.6%
	Yes	1858	83.4%
Self-medication		615	27.6%
Confidence in diabetes management	Concerned	1152	51.7%
	Confident	1076	48.3%
Awareness of diabetes care	Unaware	1367	61.4%
	Aware	861	38.6%

**Figure 2:**
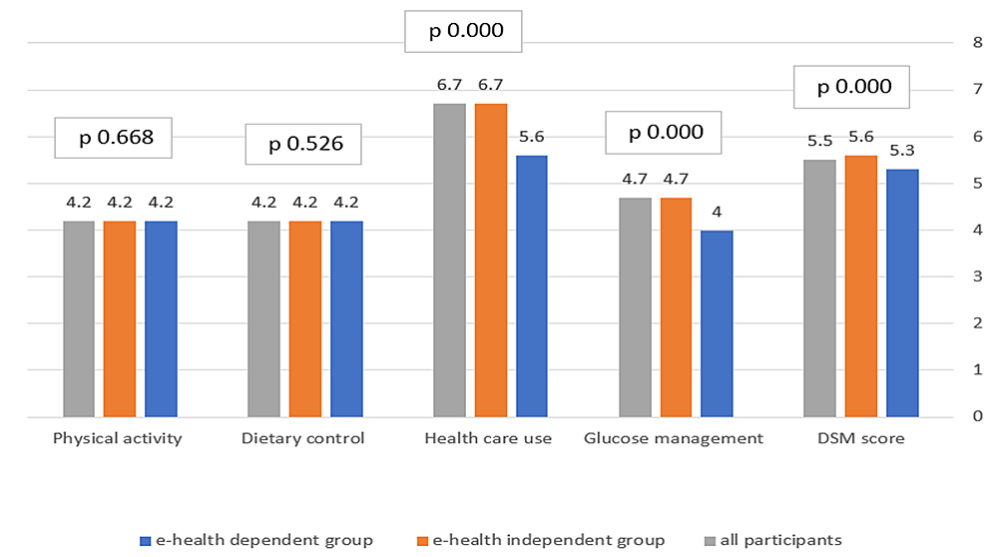
Diabetes self-management care items (n = 2,228)

The participants' DSM score is shown in Figure 2, where they had an average score of 5.2/10. They had low scores in dietary (4.2/10), physical activity (4.2/10), and glucose management (4.7/10), with high scores in health care use (6.7/10).

Diabetics with eHealth dependency showed significantly lower total DSM (5.6 vs. 5.3; p = 0.000), especially because of the significantly lower health care use (6.7 vs. 5.6%; p = 0.000) and glucose management (4.7 vs. 4.0; p = 0.000) compared to the independent group (Figure 2).

Table 2 shows that 78.1% of the participants were using eHealth, 60.1% used it regularly, and 10.2% daily. The most frequent methods were Google (55.7%) followed by social media (12.9%), and the most frequent site was the SEHA app (54.7%). Most diabetics (82%) were like to discuss the eHealth information with their physicians; while only 34.5% had an online access to their physicians. Among diabetics, many found that eHealth information is of good (25.3%) or even excellent (41.8%) quality. Several of them were satisfied (16.5%) or very satisfied (55.1%) with eHealth to the extent of replacing physician consultation (44.2%), and they were considered the eHealth-dependent group (Table 2).

**Table 2:** Frequency of using eHealth among the participating diabetic patients (n = 2,228)

		N	%
Use of eHealth	No	488	21.9%
	Yes	1339	60.1%
	Occasionally	401	18.0%
eHealth sources	Google	1242	55.7%
	Social Media	288	12.9%
	YouTube	194	8.7%
	All	21	0.9%
eHealth use frequency	Once/month	1263	56.7%
	Once/week	238	10.7%
	Daily	227	10.2%
Main eHealth portals	SEHA app	1218	54.7%
	SEHA web	233	10.5%
	WHO web	157	7.0%
eHealth quality	Fair	152	6.8%
	Good	931	41.8%
	Excellent	665	29.8%
eHealth information discussed with physician	No	401	18.0%
	Yes	1827	82.0%
Online communication with physician office	No	1459	65.5%
	Yes	769	34.5%
Online communication with hospital	Appointment	341	15.3%
	Prescription renewal	117	5.3%
	Consultation	203	9.1%
	Medical report	115	5.2%
Satisfaction with eHealth	Not	480	21.5%
	Borderline	152	6.8%
	Satisfied	368	16.5%
	Very satisfied	1228	55.1%
eHealth information is enough to replace physician visits	eHealth independent group	1243	55.8%
	eHealth-dependent group	985	44.2%

There are many factors underlying eHealth dependency, as shown in Table 3. Predictors were the region of residency (OR: 0.911; 95% CI: 0.852-0.974; p = 0.006), smoking (OR: 0.765; 95% CI: 0.626-0.935; p = 0.009), physician visit within 12 months (OR: 1.305; 95% CI: 1.061-1.605; p 0.012), frequency of care visits (OR: 1.136; 95% CI: 1.006-1.283; p = 0.040), awareness of diabetes care (OR: 0.720; 95% CI: 0.597-0.869; p = 0.001), online access to physicians (OR: 0.573; 95% CI: 0.473-0.694; p = 0.000), discussion of eHealth information with physicians (OR: 0.648; 95% CI: 0.506-0.830; p = 0.001), self-medication (OR: 0.459; 95% CI: 0.370-0.569; p = 0.000), concern about treatment (OR: 0.635; 95% CI: 0.513-0.787; p = 0.000), DSM (OR: 1.022; 95% CI: 1.006-1.039; p = 0.007), and eHealth satisfaction (OR: 0.889; 95% CI: 0.822-0.962; p = 0.003).

**Table 3:** Factors underlying eHealth dependency among the study participants *Statistically significant p-value ≤ 0.05.

	OR	95% CI		p-Value
		Lower	Upper	
Region of residence	0.911	0.852	0.974	0.006*
Diabetes duration	1.007	0.944	1.074	0.828
Age	1.011	0.901	1.134	0.855
Smoking	0.765	0.626	0.935	0.009*
Anti-diabetics	1.023	0.939	1.113	0.606
Lifestyle modification	0.862	0.668	1.111	0.251
Visit physician within 12 months	1.305	1.061	1.605	0.012*
Site of consultation	0.972	0.793	1.191	0.784
Frequency of visits	1.136	1.006	1.283	0.040*
Awareness of diabetes care	0.720	0.597	0.869	0.001*
Online access to physicians	0.573	0.473	0.694	0.000*
Discussion of eHealth information	0.648	0.506	0.830	0.001*
Satisfaction with physician diabetes care	0.897	0.775	1.039	0.147
Self-medications	0.459	0.370	0.569	0.000*
Concern about diagnosis	0.906	0.730	1.125	0.371
Concern about treatment	0.635	0.513	0.787	0.000*
eHealth satisfaction	0.889	0.822	0.962	0.003*
Diabetes self-management	1.022	1.006	1.039	0.007*

## Discussion

This study shows that approximately three-quarters of T2DM patients use eHealth information, with 44.2% of them depending on information gained online to the extent of replacing the role of physicians. The primary sources of eHealth were Google and social media, especially from the "SEHA" application. The major factors associated with eHealth dependency were physician visits, discussion, and online communication and satisfaction with eHealth quality. Other factors included patient concerns, self-medication, and management. Unfortunately, the eHealth-dependent patients showed significantly lower DSM scores, especially with regard to the use of health care services and glucose management compared to the other independent groups.

A large number of eHealth users in this study is much higher than that reported by another Saudi study in Riyadh, where only 27.9% of the study participants were seeking diabetic information online. However, the results of this study were in parallel with the recent overall increase in Internet usage in Saudi Arabia [13] and other countries [21].

A substantial number of patients considered eHealth as a substitute for doctors' visits. Similarly, others reported that the use of eHealth may postpone or replace medical consultations [22] in up to 30% of patients [23]. This trend is seen well in young patients [24], probably because of their ability to use electronic options. However, this trend was not observed in the present study. There are many possible interactions between the use of eHealth and the health care services provided by the diabetes care team. The use of eHealth might be linked to a lower number of regular physician visits [13], as in our patients. On the contrary, information gained from the Internet might influence patients to seek medical advice, help them to discuss many important issues during their visit, or guide them after the visit for more information on emerging topics [25].

Lower use of health care services with lower glucose management was reported in our study among the eHealth-dependent group. These findings are inconsistent with another study that reported that people in poor health are more likely to seek disease-related information online and use health care services to a larger extent [26]. eHealth use is supposed to be a fundamental part of DSM by providing easily accessible patient education with a consequent reduction in diabetes education costs and burden [27].

Our results suggest that despite the increasing consumption of eHealth among diabetics, they still need to discuss their concerns about their disease with their physicians. Moreover, the dependency on eHealth in this study was linked to the DM patients' Internet satisfaction but not to their satisfaction with the services provided by the health care professionals. This could reflect continued trust in the health system. These results are concurrent with those of previous reports [28]. Therefore, health care providers need to maintain continuity of and access to remote care and consultation with their patients. This will open the door to the use of telemedicine options in diabetes care. Telemedicine has evolved over the last decades to improve the accessibility and quality of care among patients and health care providers and to solve health care access challenges, especially during crises, such as during the COVID-19 pandemic.

The main limitation of this study is the use of a cross-sectional study design, which, together with a voluntary response sampling technique, will limit the generalization of its results to all Saudi T2DM patients. Moreover, the arbitrary labeling of the eHealth-dependent group based on the answer to one question is another limitation of this study.

## Conclusions

Most Saudi T2DM patients with average DSM use different eHealth resources and are satisfied with their quality. The dependency of eHealth is significantly associated with lower DSM, especially for health care use and glucose monitoring management, a finding that could affect patient outcomes. Still, patients need to communicate with their physicians in person, who should have different options for remote consultation, such as telemedicine, to support their patients.
